# MicroRNA Alterations and Associated Aberrant DNA Methylation Patterns across Multiple Sample Types in Oral Squamous Cell Carcinoma

**DOI:** 10.1371/journal.pone.0027840

**Published:** 2011-11-22

**Authors:** Erik D. Wiklund, Shan Gao, Toby Hulf, Tennille Sibbritt, Shalima Nair, Daniela Elena Costea, Sune B. Villadsen, Vivi Bakholdt, Jesper B. Bramsen, Jens A. Sørensen, Annelise Krogdahl, Susan J. Clark, Jørgen Kjems

**Affiliations:** 1 Department of Molecular Biology and Genetics, Aarhus University, Aarhus, Denmark; 2 Epigenetics Group, Cancer Program, Garvan Institute of Medical Research, Darlinghurst, New South Wales, Australia; 3 Section for Pathology, The Gade Institute, University of Bergen, Bergen, Norway; 4 Department of Plastic Surgery, Odense University Hospital, Odense, Denmark; 5 Department of Pathology, Odense University Hospital, Odense, Denmark; East Carolina University, United States of America

## Abstract

**Background:**

MicroRNA (miRNA) expression is broadly altered in cancer, but few studies have investigated miRNA deregulation in oral squamous cell carcinoma (OSCC). Epigenetic mechanisms are involved in the regulation of >30 miRNA genes in a range of tissues, and we aimed to investigate this further in OSCC.

**Methods:**

TaqMan® qRT-PCR arrays and individual assays were used to profile miRNA expression in a panel of 25 tumors with matched adjacent tissues from patients with OSCC, and 8 control paired oral stroma and epithelium from healthy volunteers. Associated DNA methylation changes of candidate epigenetically deregulated miRNA genes were measured in the same samples using the MassArray® mass spectrometry platform. MiRNA expression and DNA methylation changes were also investigated in FACS sorted CD44^high^ oral cancer stem cells from primary tumor samples (CSCs), and in oral rinse and saliva from 15 OSCC patients and 7 healthy volunteers.

**Results:**

MiRNA expression patterns were consistent in healthy oral epithelium and stroma, but broadly altered in both tumor and adjacent tissue from OSCC patients. MiR-375 is repressed and miR-127 activated in OSCC, and we confirm previous reports of *miR-137* hypermethylation in oral cancer. The miR-200 s/miR-205 were epigenetically activated in tumors *vs* normal tissues, but repressed in the absence of DNA hypermethylation specifically in CD44^high^ oral CSCs. Aberrant miR-375 and miR-200a expression and *miR-200c-141* methylation could be detected in and distinguish OSCC patient oral rinse and saliva from healthy volunteers, suggesting a potential clinical application for OSCC specific miRNA signatures in oral fluids.

**Conclusions:**

MiRNA expression and DNA methylation changes are a common event in OSCC, and we suggest miR-375, miR-127, miR-137, the miR-200 family and miR-205 as promising candidates for future investigations. Although overall activated in OSCC, miR-200/miR-205 suppression in oral CSCs indicate that cell specific silencing of these miRNAs may drive tumor expansion and progression.

## Introduction

Oral squamous cell carcinoma (OSCC) is the sixth most common human malignancy worldwide, and the incidence rate is increasing, especially in younger people [1], [2]. Over 400,000 individuals die from this disease every year, with 80% of deaths occurring in developing countries [3]. Oral carcinogenesis is believed to represent a multi-step process driven by the accumulation of carcinogen-induced genetic changes, and it is generally accepted that many of the cancer-associated gene changes stem from the gain, loss or mutation of genetic information [4]. However, it has become increasingly clear that epigenetic events, heritable changes in gene regulation not related to changes in the primary DNA sequence, may also play an important role in development of cancer [5]. Many findings have thus shown that human cancer, including OSCC, is not only a polygenetic, but also a polyepigenetic disease [6], [7].

The most frequent and readily detectable epigenetic event in cancer is DNA hypermethylation of tumor suppressor genes, with hypomethylation associated activation of oncogenes being being less observed phenomenon [5], [8]. CpG island hypermethylation in human cancer has been shown for tumor suppressor, DNA repair, hormone receptor, angiogenesis inhibitor and non-coding RNA (ncRNA) genes [5]. In OSCC, silencing of *p16^INK4a^*, *DAPK1*, *ABO*, *MGMT*, and *CDH1* has been linked to increased DNA methylation of the respective promoters, with frequencies varying between 20% and 80% of oral primary tumors [9], [10], [11]. Interestingly, Hasegawa *et al.* [12] found that silencing of E-cadherin by *CDH1* CpG island methylation is related to smoking, thus increased cancer risk in smokers may in part be a result of tobacco induced epigenetic effects.

Moreover, the expression of microRNAs (miRNAs), a class of ∼22 nt non-coding RNAs that regulate gene expression post-transcriptionally [13], is broadly altered in most types of cancer [14]. Many miRNAs are involved in important developmental processes such as differentiation and epithelial to mesenchymal transition (EMT), and are therefore often highly tissue and lineage specific. MiRNA expression signatures can thus be used to classify tumor subtype and origin and predict prognosis [15]. In addition, miRNAs are relatively readily detectable compared to more unstable and variable mRNAs, and some are also secreted by exocytosis into bodily fluids, making them promising as diagnostic and prognostic markers, as well as therapeutic targets [15].

Only a small number of reports have investigated miRNA expression profiles in OSCC. Some important candidate miRNAs in oral cancer etiology and progression have been proposed, including loss of miR-34b, miR-100, miR-125b miR-137, miR-193a and miR-203, which seems to occur specifically in OSCC compared to normal oral mucosa [7], [16]. Overexpression of miR-21, miR-181b and miR-345 has been suggested as a signature of malignant transformation of leukoplakia, for which histological assessments have limited prognostic value [17]. However, and further analyses of OSCC specific miRNA profiles, with insight into the underlying regulatory mechanisms and functional consequences, are required to consolidate current observations.

MiRNA deregulation can occur at both the transcriptional and processing level, and aberrant DNA methylation patterns have been implicated in altered expression of 30 or so miRNA genes in different types of cancer [18]. A comprehensive bioinformatic analysis found that about 50% of miRNA genes are associated with CpG-islands [19], suggesting that many more miRNAs are candidate targets of the DNA methylation machinery. There has only been one extensive study of epigenetic silencing of miRNA genes in oral cancer, which identified that repression of miR-34b, miR-137, miR-193a and miR-203 is associated with increased DNA methylation in oral cell lines and OSCC tumors [7]. Notably, increased *miR-137* DNA methylation levels was linked to poor OSCC survival rates [20], and was detected specifically in patient oral rinse [21], suggesting it might provide a readily detectable prognostic marker for OSCC.

Here, we aimed to further investigate miRNA deregulation with a possible link to DNA hypermethylation in OSCC. MiRNA expression patterns were analyzed by a combination of qPCR arrays and individual miRNA assays for a panel of 25 OSCC tumors with matched adjacent tissue and 8 control healthy oral stroma and epithelium pairs. The DNA methylation status of candidate epigenetically deregulated miRNA loci was subsequently measured using the semi-quantitative MassArray® mass spectrometry platform.

In general, both miRNA expression and DNA methylation patterns were consistent across healthy controls, but highly variable in both tumor and matched adjacent samples, thereby highlighting tumor heterogeneity. Consistent with previous reports, *miR-137* methylation was increased in OSCC tumors. MiR-375 was identified as strongly repressed and miR-127 as activated in OSCC tumors, but this regulation appeared independent of epigenetic change. There was a correlation between miR-200 family and miR-205 expression and DNA methylation levels across the sample panel, with concurrent increased expression and DNA hypomethylation in tumors compared to matched normal tissue. Interestingly, we observed an epigenetically independent loss of miR-200 and miR-205 expression in CD44^high^ cancer stem cell (CSC) populations sorted from primary OSCC samples, suggesting that these miRNAs are specifically repressed in cells driving tumor expansion and progression. Finally, we show that miR-375 and miR-200a levels are lower in OSCC patient oral rinse and saliva compared to healthy controls. This demonstrates that miRNAs are present and detectable in oral fluids, and could potentially be translated into non-invasive OSCC clinical tests.

## Materials and Methods

### Sample preparation

#### Primary tissue samples

Frozen surgical OSCC specimens from 25 patients were obtained from Odense University Hospital, Denmark. The median age was 61 years (range 48–94 years), and there were 9 females and 16 males. A tumor adjacent clinically normal mucosa biopsy was also collected from all patients. Hematoxylin-eosin staining of the histological sections was performed to examine the ratio between cancer and normal tissues. In 20 cases the cancer cells contributed 80% or more; these tissue blocks were used directly for RNA preparation. In the 5 cases with lower tumor load, a laser microdissection system (P.A.L.M.) was used to separate cancer cells from adjacent normal tissue. Buccal mucosa samples were obtained from 8 healthy volunteers (5 females and 2 males, median age 32 years, range 25–62 years). To facilitate separation of healthy epithelium and stroma, the healthy normal tissue samples were incubated in 2 mM EDTA buffer, as previously described by MacKenzie *et al.* [22]. Informed written consent was obtained individually and the research was approved by the Ethics Committee in Region Middle Jutland according to Danish legislation (M-20110028). The full name of the ethics committee is “The Central Denmark Region Committees on Biomedical Research Ethics” (Danish name is “De Videnskabsetiske Komitéer for Region Midtjylland”, homepage: www.komite.rm.dk).

#### Oral rinse (Buccal cells) collection

Careful brushing of teeth and abstinence from food and drinking was required 1 hour before sample collection. The mouth was cleaned by water and subsequently rinsed with 10 ml PBS for 30–60 seconds. PBS collection was repeated 4 times. The 40 ml total solution was divided into 2 portions, centrifuged at 10,000 rpm for 10 min, and the supernatant removed. One pellet was snap frozen at −80°C for DNA purification and the other was dissolved in 1 ml RNA later reagent (Qiagen, Copenhagen), kept at room temperature for 24 hours, and then stored at −20°C until RNA purification.

#### Saliva collection

Immediately after mouthwash collection, ∼2 ml saliva was collected and divided into 2 portions. One was snap frozen at −80°C for DNA purification. The other portion was mixed with 5 ml RNA Protect Saliva reagent (Qiagen, Copenhagen), kept at room temperature for 24 hours, and then stored at −20°C until RNA purification.

Oral rinse and saliva were collected totally from 15 OSCC patients and 7 healthy volunteers.

### RNA and DNA isolation

Total RNA from primary samples, oral fluids and cell lines was purified using either TRIzol® RNA extraction reagent (Invitrogen, Copenhagen) or the RNeasy® kit (Qiagen, Copenhagen) according to supplier's protocol and stored at −80°C. RNA quality was examined on a 2100 Bioanalyzer® chip (Agilent, Santa Clara CA), and only samples with RIN >7 were used for TLDA analysis. DNA from primary samples and oral fluids was column-purified using the DNeasy® blood & tissue kit (Qiagen, Copenhagen) according to the supplied protocol and stored in aqueous solution at −20°C.

### MiRNA expression analyses

MiRNA expression profiling was performed using the TaqMan® low density array (TLDA) qRT-PCR system (Applied Biosystems, Foster City CA). TLDA v1.0 Early Access assays for 368 mature miRNAs, and was performed as per manufacturer's protocols. In brief, 0.5–1 µg total RNA from primary samples were reverse transcribed with pools of miRNA specific RT-primers, and the resultant cDNA was subjected to real-time reverse transcription quantitative qRT-PCR using specific forward primers and TaqMan® fluorescent probes in an array format. RNU48 was selected for normalization based on current literature [23] and our own experimental validations, indicating that RNU48 was the most reliable reference with consistent expression for all samples (Fig. S1). Initial analyses were performed using SDS software (Applied Biosystems). Statistical tests for changes in expression (via Cts) were performed using moderated t-tests without multiple testing corrections using the R limma package [24]. Mature miRNAs were deemed differentially expressed at p<0.05 and a −fold change of ≥1.5. Agglomerative hierarchical clustering of the miRNA expression profiles was performed using the Cluster 3.0 software, requiring a miRNA to be detected in a minimum of 10 samples to be included.

Specific miRNA expression was determined by miRNA TaqMan® qRT-PCR assays (Applied Biosystems) according to supplier's protocol using 10 ng total RNA, and run in triplicates on a LightCycler® 480 (Roche, Basel Switzerland). As for the TLDA profiling, expression was normalized to RNU48 (2^−ΔCt^) (TaqMan® assay, Applied Biosystems) or miR-191 for oral fluid samples. The latter was chosen because miR-191 is secreted and robustly detectable in 12 different body fluids [25].

### CpG cluster prediction

Regions significantly enriched for CpG dinucleotides were predicted using the online sliding window algorithm CpGPlot in the EMBOSS package from EMBL-EBI [26] (window = 100 nt; step = 1; Obs/Exp = 0.6; MinPC = 50; Length = 50/100).

### DNA methylation analysis

Bisulphite conversion of DNA was performed as described by Clark *et al*. [27], using 0.1–2 µg DNA purified from primary tissue and oral fluids. Bisulphite specific primers were designed by standard principles for PCR amplicons in the 200–400 bp range (Table 1), adding forward and T7 promoter reverse primer tags required for Sequenom analysis according to specified requirements in [27]. PCR was carried out in duplicate at an annealing temperature of 55°C, 40 cycles, using Platinum® Taq DNA polymerase (Invitrogen). DNA methylation analysis of pooled duplicate bisulfite PCR amplicons for all primary and oral fluid samples was performed using the MassArray® MALDI-TOF mass spectrometry platform (Sequenom, San Diego CA) according to supplier's protocol and improvements described by Coolen *et al*. [28]. The DNA methylation level was scored as percentage methylation of individual CpG units (comprising one or more CpG-sites) averaged across the full CpG regions, thus arriving at an average percentage miRNA locus DNA methylation for each sample.

**Table 1:** Bisulphite primers.

Name	Fwd primer	Re primer	Amplicon size
miR-34b	TTYGGTTTGTAGGTAGTGTTATTAGT	CRAAACAAACTACRCAAA	194
mir-127	GAAGTTTAGAGGGTTTTGATTT	ACCCCTAATATATATCCACCAA	356
miR-137	TGAAAAGAATAAGAAAGTGTTATTTTGGTA	AACTCAACCCATCCCCAAAC	309
miR-155	GGGTGTTTTTTGGGGATTTAGT	AAAAACAATCTCTTTTTCCACCC	249
miR-200ab-429	TTATGGGAGTTTAGGGGATATATT	ATTCAAACCTACACAAATAAA	224
miR-200c-141	AAGGTTATTAGGGGAGAGGTTT	CTTCAAACCCAAAATCCCTA	313
miR-203	GTYGGGTGGTTGTAGTAGGGT	ACCCCTAACTATAACTCTAACTCCAAA	295
mir-205	GAGTGAAGTTTAGGAGGTATGGAGT	CACTCCAAATATCTCCTTCATTA	189
miR-375	GTGATTTTTTGGTTTTGGTTTTT	AAAACTAAACAAACAATATAAAAACACAC	277
mir-410	GGGAGGGTTTTTTGTAAGTAT	CTCCCTTCAAATACCAAAAAAA	376

### Cell culture

All oral cell lines were obtained as a gift from Professor Erik Dabelsteen, School of Dentistry, University of Copenhagen. Cells were maintained in Dulbecco's modified Eagle's medium (DMEM) supplemented with 10–20% fetal calf serum (Invitrogen), 50 units/ml penicillin and 50 mg/ml streptomycin (both Sigma-Aldrich, St. Louis MO) under standard cell culture conditions (37°C, 5% CO2). For the DNA demethylation and histone deacetylatse inhibition experiments, culture cells were treated with 1–3 µM 5-aza-2'deoxycytidine (5-Aza-dC) for 48 h or 3 µM 5-Aza-dC for 24 h followed by 50 nM trichostatin A (TSA) for 24 h (both Sigma-Aldrich), as described in Hinshelwood *et al* 2007 [29].

### Statistical analyses

Statistical significance was determined by the Student's t-test using Microsoft Excel® 2004. The paired test was used to assess significance between tumor/matched adjacent normal and epithelium/stroma pairs, and the unpaired unequal variance Welch's t-test for patient vs. healthy control tissues.

## Results

**Figure 1:**
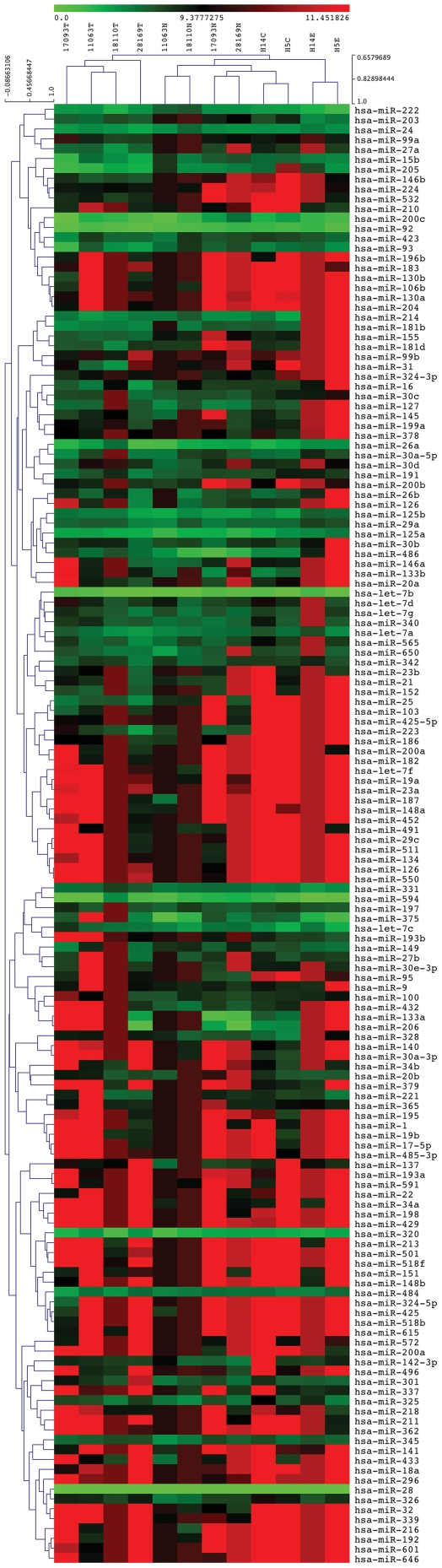
Hierarchical clustering of OSCC and healthy oral tissue miRNA expression profiles. MiRNA expression profiling was performed using TaqMan® TLDA low density miRNA arrays for primary tumor/matched normal adjacent pairs from 4 OSCC patients, as well as normal epithelium and stroma from 2 healthy controls. The samples encompass 2 non-metastatic tumors (18110T & 11063T), 2 metastatic tumors (17093T & 28169T), 4 matched adjacent normal tissues (18110N, 11063N, 17093N & 28169N), 2 healthy connective tissue (stroma) (H14C & H5C) and 2 healthy epithelium (H14E & H5E), respectively. Expression was normalized to RNU48 (2^−dCt^) and hierarchical clustering was performed using Cluster 3.0. for relative miRNA levels, and is color coded from bright green (undetected) to red (highest expression).

As an initial screen for deregulated miRNAs, we performed TaqMan® miRNA low density array (TLDA) analysis for 2 non-metastatic and 2 lymph node metastasis tumor and matched adjacent epithelium pairs from OSCC patients, as well as normal oral epithelium and stroma from 2 healthy controls. Hierarchical clustering of the miRNA profiles showed that non-metastatic (patients 18110 & 11063) and metastatic (patients 17093 & 28169) tumors, adjacent normal and healthy control tissues fall into separate groups, indicating that a specific miRNA pattern is associated with OSCC (Fig. 1). The miRNA signatures were most broadly altered between OSCC tumors and healthy epithelium, with the matched adjacent tissue and stroma clustering in between. As such, although of epithelial phenotype, OSCC tumors appear more distinct from their origin than endogenous differences in oral epithelium and connective tissue, thus highlighting the comprehensive miRNA changes involved.

Epigenetically deregulated miRNAs in OSCC were subsequently predicted based on the observed expression patterns, the presence of CpG elements nearby the respective miRNA loci and current literature. This initial list of possible candidates consisted of miR-34b, miR-127, miR-155, the miR-200 family (miR-200abc, miR-141 and miR-429), miR-203, miR-205, miR-375 and miR-410. Although miR-137 expression was below the detection limit for all samples in our initial screening, this was also included as it has previously been associated with DNA hypermethylation in OSCC [7], [20].

**Figure 2:**
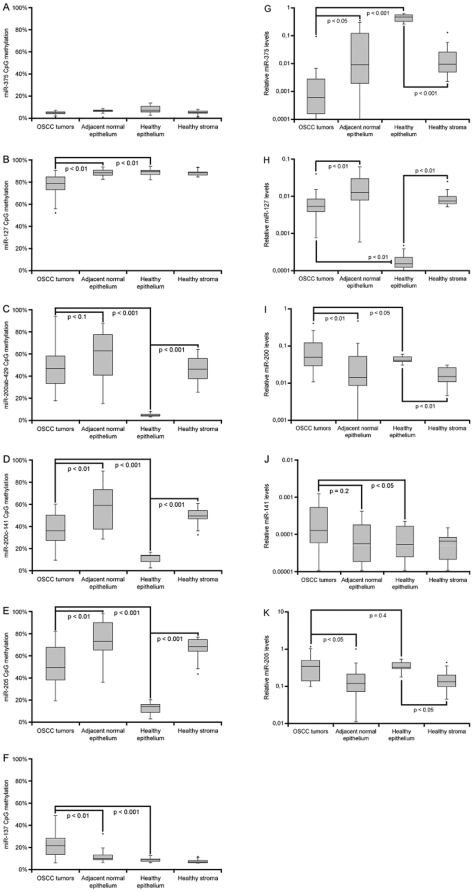
DNA methylation and expression patterns for candidate epigenetically deregulated miRNAs. MiRNA CpG methylation (**a–f**) and expression (**g–k**) patterns in OSCC in and normal oral tissues. DNA methylation levels were measured using the MassArray® mass spectrometry platform for CpG enriched regions in the *miR-375* (**a**), *miR-127* (**b**), *miR-200ab-429* (**c**), *miR-200c-141* (**d**), *miR-205* (**e**) and *miR-137* (**f**) loci in a panel of 25 OSCC tumor/matched adjacent pairs and 8 healthy control epithelium/stroma pairs. Values are presented as average percentage CpG methylation across the full regions analyzed for each locus. Relative expression of miR-375 (**g**), miR-127 (**h**), miR-200abc (average) (**i**), miR-141 (**j**) and miR-205 (**k**) was evaluated in the same samples by individual TaqMan® miRNA assays performed in triplicates and normalized to RNU48 (2^−dCt^). Box plots indicate the median value (horizontal line) and the 25^th^–75^th^ percentile range (box) with whiskers at 1.5×IQR. Values outside this range are shown as outliers (points). P-values were determined by the paired two-tailed Student's t-test for matched patient tumor/adjacent and healthy epithelium/stroma pairs overall, and the unpaired two-tailed Student's t-test for tumor vs. healthy epithelium.

The DNA methylation level of the respective miRNA loci was assessed using the MassArray® mass spectrometry platform for an expanded panel of primary samples, consisting of 25 tumor/matched normal and 8 healthy stroma/epithelium pairs (Fig. 2). A broad spectrum of CpG methylation patterns were observed, ranging from completely methylated to unmethylated, thus demonstrating the dynamic range of the MassArray® system. Statistically significant differential methylation was observed between tumor and matched normal and/or healthy epithelium for the *miR-127*, *miR-137*, *miR-200ab-429*, *miR-200c-141* and *miR-205* loci (Fig. 2), but no distinct differences were observed between metastatic and non-metastatic tumors. *MiR-375* remained unmethylated across the sample panel (Fig. 2a), and *miR-34b*, *miR-155*, *miR-203* and *miR-410* displayed unchanged or inconsistent DNA methylation patterns (data not shown).

Having established a shorter list of epigenetically deregulated candidates (miR-127, miR-137, the miR-200 family and miR-205), we went on to measure their expression level in the full sample panel by individual TaqMan® miRNA assays. As the most strongly repressed miRNA, miR-375 was also included (Fig. 2). Similar to DNA methylation, miRNA expression patterns were highly variable in the OSCC tumors and matched adjacent tissues, but consistent in healthy normal epithelium and stroma. This further illustrates broad changes in both miRNA expression and DNA methylation in OSCC patients. Again, there were no clear differences between metastatic and non-metastatic tumors for these miRNAs. MiR-375 was confirmed as a strongly repressed miRNA in OSCC, with 10 −fold decreased expression in tumors compared to matched adjacent tissue, and a 1000 −fold change when comparing to healthy epithelium (Fig. 2g). This miRNA is probably epithelial specific, highlighted by 2 orders of magnitude higher miR-375 expression in healthy epithelium than stroma. MiR-137 remained undetected in all samples, suggesting that mature miR-137 is silenced in oral tissues regardless of *miR-137* CpG methylation status (data not shown).

The expression patterns of miR-127, the miR-200s and miR-205, on the other hand, were more complex (Fig. 2h–k). Mature miRNA levels were distinctly different in healthy oral epithelium and stroma, reflective of miRNA tissue specificity. Although a significant change was observed between tumor and matched normal pairs, the adjacent tissues were overall more similar to stroma than epithelium, indicating that both tumors and adjacent epithelium may gain stromal-like features in OSCC patients (Fig. 2h–k). MiR-127 appears to be a stroma specific miRNA, and is present at lower levels in OSCC tumors than in corresponding adjacent tissue, but overall upregulated in patients compared to healthy epithelium. Consistent with previous reports, the miR-200-family and miR-205 are most active in epithelium, and their expression is increased in OSCC tumors compared to matched adjacent normal (Fig. 2h–k) [30], [31], [32].

Neither in the healthy control tissues, nor in the tumor and matched normal pairs, could changes in miR-127 expression be accounted for by corresponding DNA methylation patterns, which remained elevated throughout the sample panel. Rather, miR-127 repression was associated with a seemingly contradictory statistically significant pair-wise loss of methylation in tumor vs. adjacent tissue (Fig. 2b). This phenomenon has also been observed in prostate cell lines [33]. However, as the locus remains 75–80% methylated, this might not affect transcription levels and could merely be a result of broad, unspecific CpG methylation changes in the tumors.

**Figure 3:**
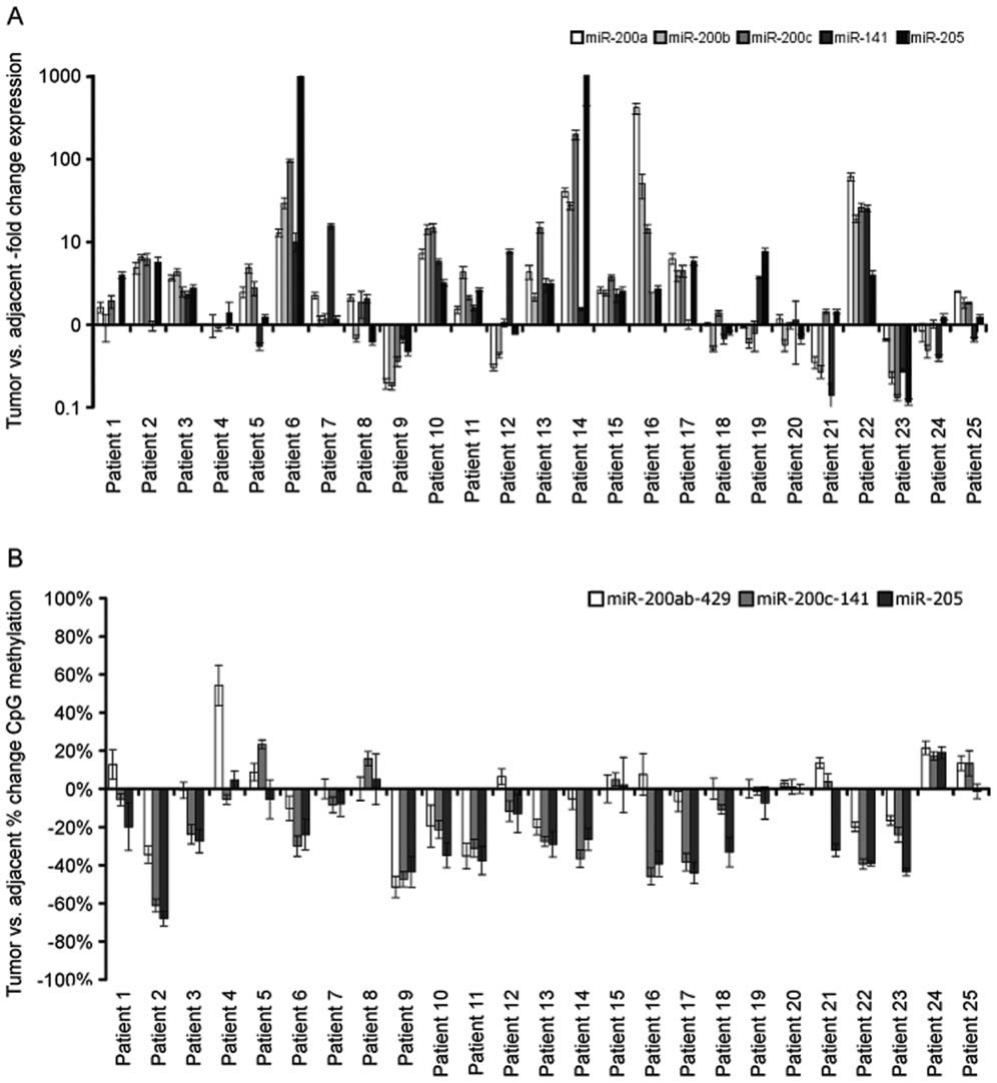
miR-200 and miR-205 expression and methylation differences in individual tumor vs. matched adjacent pairs. Expression and DNA methylation was determined as explained in the legend to Figures 2, and represents the same data visualized as the difference between individual pairs of tumor and matched adjacent epithelium. (**a**) miR-200a, miR-200b, miR-200c, miR-141 and miR-205 −fold change expression (+/− SD), and (**b**) average % CpG methylation change of the *miR-200ab-429*, *miR-200c-141* and *miR-205* loci (+/− SE). X-axis numbers are OSCC patient identifiers.

For the miR-200s and miR-205, however, expression and methylation patterns were indicative of a role for epigenetic regulation. In healthy controls, high expression correlated with low DNA methylation in epithelium, with the reciprocal pattern observed in stroma. Figure 3 highlights by pair-wise comparison that activation of miR-200/miR-205 is strongly correlated with overall decreased CpG methylation of the *miR-200ab-429*, *miR-200c-141* and *miR-205* loci in OSCC patients. Thus, in line with previous reports in cell lines and bladder and breast cancer [34], [35], [36], the miR-200 family and miR-205 appears to be coordinately epigenetically regulated in healthy and diseased oral tissues.

Activation of the miR-200 family and miR-205 has also been reported in other forms of cancer, however, their expression is less prominent in invasive and metastatic tumors. This can be explained by miR-200/205 targeting ZEB1 and ZEB2, which are transcriptional repressors of E-cadherin and master regulators of EMT [30], [31]. In addition, miR-200/miR-205 repression leads to activation of Notch signaling [37], and miR-200c also targets BMI1, a regulator of stem cell renewal [35], [38]. Reactivation of miR-200c inhibits stem cell directed expansion of breast tumors in mice [35], and miR-200c expression is low in head and neck squamous cancer stem cells (CSCs) [38]. Repression of the miR-200s and miR-205 therefore leads to silencing of E-cadherin and BMI1, followed by EMT, loss of cell anchorage and cellular dedifferentiation, probably inducing CSC formation [32], [35], [39]. To investigate a broader role of the miR-200 family and miR-205 regulation in OSCC, we therefore went on to investigate their regulation in oral CSC populations.

**Figure 4:**
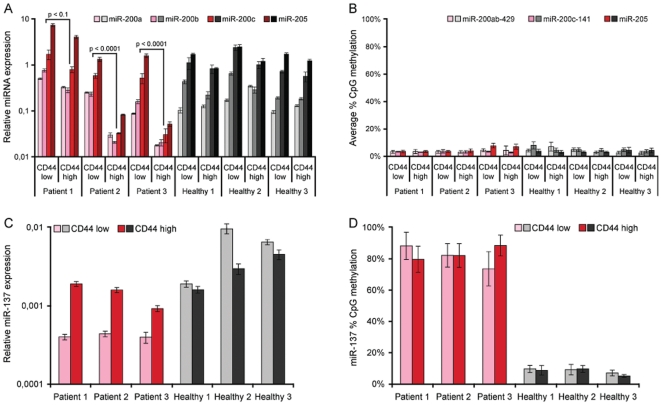
miRNA expression and DNA methylation in oral cancer stem cells. MiR-200 family and miR-205 expression (**a**) and DNA methylation (**b**), and miR-137 expression (**c**) and DNA methylation (**d**) in OSCC cancer stem cells (CSCs) purified by FACS sorting the 5% most CD44 staining cells from 3 primary samples (CD44^high^), compared to the 5% least staining cells (CD44^low^). Similar control non-CSC cells were sorted from 3 healthy individuals. Relative miRNA expression (+/− SD) was determined by individual TaqMan® miRNA assays normalized to RNU48 (2^−dCt^), and percentage CpG methylation levels (+/− SE) across the indicated regions were measured using the MassArray® mass spectrometry platform. Statistical significance was determined by the paired one-tailed Student's t-test for CD44^high^ vs. CD44^low^ cells from individual patients.

The CSC hypothesis entails that a subpopulation of dedifferentiated stem-like cells drive tumor expansion and progression. These cells have elevated levels of the cell surface marker CD44 in a variety of cancers, including OSCC [38], [40]. Here, oral CSCs were enriched by FACS sorting the top 5% of CD44-positive cells (CD44^high^) from 3 primary tumors. As reference, the bottom 5% CD44-negative cells (CD44^low^) and populations from healthy controls, were also isolated. Strikingly, miR-200 and miR-205 expression was 2–10 −fold lower in tumor CD44^high^ CSCs compared to the CD44^low^ fraction, whereas no difference was observed between healthy control CD44^high^ and CD44^low^ populations (Fig. 4a–b). This CD44^high^ specific miR-200/miR-205 loss was not associated with increased DNA methylation. It thus seems that, although overall activated in OSCC tumors, miR-200 and miR-205 expression is specifically repressed independently of epigenetic silencing in tumor driving CD44^high^ oral CSCs.

MiR-137 has also been linked to stem cell differentiation by a number of studies [41], [42], [43], therefore we investigated expression and DNA methylation of this miRNA in the CD44 sorted populations as well (Fig. 4c–d). Unlike for the primary tissue samples, in these purified populations mature miR-137 was readily detectable. Expression was lower in cancer compared to normal, with corresponding *miR-137* CpG methylation levels of ∼80% and <10%, respectively. This confirms previous reports of epigenetic miR-137 silencing in OSCC, but suggests that this occurs in specific cell types and may not be detectable in total, heterogenous tumor samples. Moreover, an increase in miR-137 expression, independently of altered DNA methylation, was observed in patients', but not healthy CD44^high^ compared to CD44^low^ cells. In fact, cancer and normal CD44^high^ cells displayed relatively similar miR-137 expression, with the greatest difference between the CD44^low^ populations (Fig. 4c–d). The main miR-137 deregulation may thus not occur in tumor and progression driving CSCs. In addition, as for miR-200/miR-205, epigenetic mechanisms can only partly account for miR-137 repression in OSCC.

**Figure 5:**
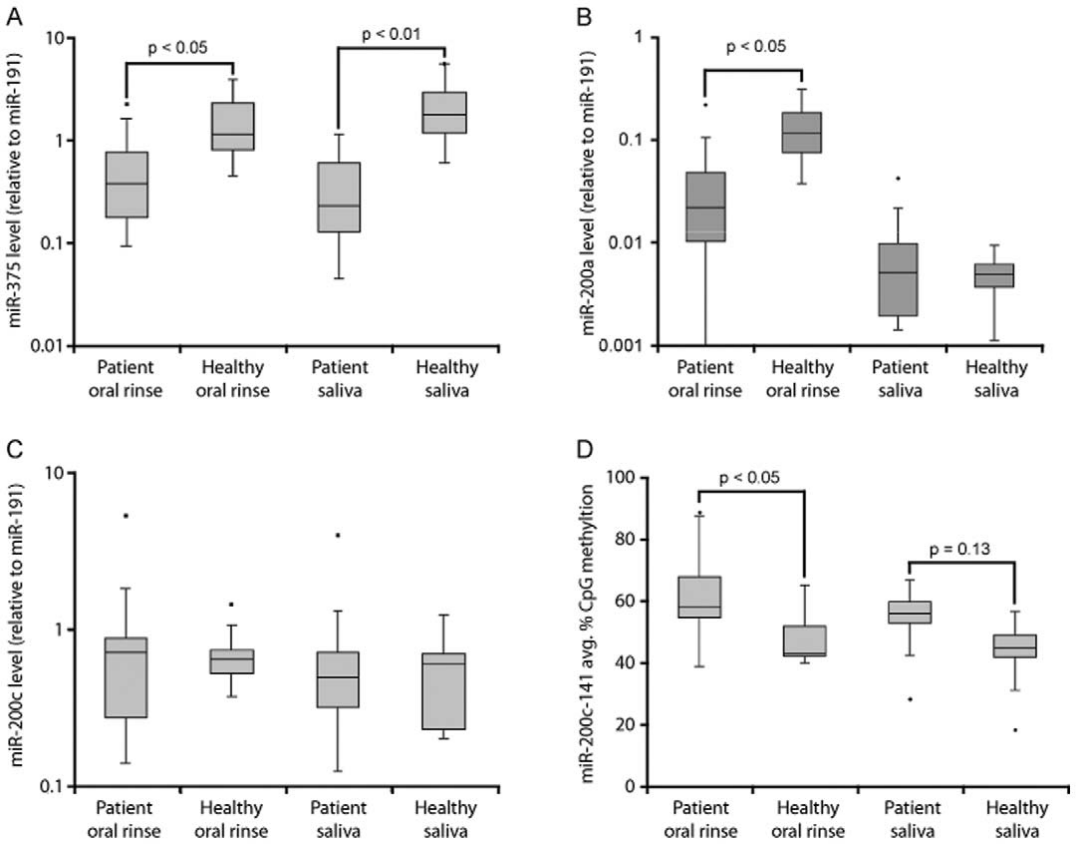
miRNA expression and DNA methylation in oral rinse and saliva. (**a**) miR-375 (**b**) miR-200a and (**c**) miR-200c relative to miR-191 expression ratio, and (**d**) absolute *miR-200c-141* DNA methylation level in oral rinse and saliva from 15 OSCC patients and 7 healthy controls. The box plots represent the median value (horizontal line) and the 25^th^–75^th^ percentile range (box) with whiskers at 1.5×IQR. Values outside this range are indicated as outliers (points). MiRNA ratio (+/− SD) was determined by individual TaqMan® miRNA assays, normalizing miR-375 to miR-191 Ct values (2^−dCt^). Percentage CpG methylation (+/− SE) was measured using the MassArray® mass spectrometry platform. P-values were determined by the unpaired two-tailed Student's t-test for OSCC patients vs. healthy controls.

Finally, in order to investigate possible diagnostic relevance, we tested whether any of the OSCC specific miRNA expression and/or DNA methylation changes could be used to distinguish patient and control oral fluids. Mature miRNA and DNA methylation levels were assessed in RNA and DNA isolated from oral rinse and saliva from 15 OSCC patients and 7 healthy subjects (Fig. 5). When measuring secreted miRNA levels, it is no longer suitable to use intracellular snoRNA (RNU48) for normalization. Therefore, we selected miR-191 as a reference for profiling oral rinse and saliva samples based on the notion that this miRNA is secreted into a variety of body fluids and is considered a robust miRNA for qPCR normalization purposes [25], [44]. Consistent with repression in tumors, miR-375 levels relative to miR-191 were lower in both patient oral rinse and saliva (Fig. 2g & Fig. 5a). MiR-200a relative to miR-191 levels were also decreased in patient oral rinse, but remained unchanged in saliva (Fig. 5b).

Whereas miR-200c levels were stable across all oral fluid samples, *miR-200c*-*141* CpG methylation was significantly elevated in patient oral rinse, but not in saliva. This is consistent with the generally higher methylation of this locus observed for tumor and matched adjacent tissue from OSCC patients compared to healthy epithelium (Fig. 2d & Fig. 5c–d). Certain OSCC specific miRNA and DNA methylation signatures are therefore both present and detectable in oral fluids, and have potentially clinical diagnostic application.

## Discussion

According to our observations, miRNA expression and corresponding DNA methylation patterns are tightly controlled in, and often specific to, healthy oral epithelium and stroma. In OSCC patients, on the other hand, these patterns are broadly altered, representing variable changes occurring in both primary tumor samples and matched adjacent tissue (Figs. 1–2). Therefore, in line with previous work [7], [16], [45], it seems that OSCC is associated with a range of aberrant epigenetic and miRNA expression patterns. We did not identify any specific changes in miRNA expression profiles or DNA methylation patterns between non-metastatic and metastatic OSCC tumors. However, as the miRNA expression profiles cluster separately based on global signatures (Fig. 1), there are probably differences in miRNA species other than those investigated in detail here, such as for instance miR-34, miR-21 and miR-203 (Fig. 1 and data not shown).

Specifically, we identify miR-375 as a highly significantly repressed miRNA in OSCC tumors compared to matched adjacent normal tissues and healthy tissues from volunteers (Fig. 2a). Loss of miR-375 has also been reported in gastric, liver and breast cancers, and a putative tumor suppressor role has been linked to a failure to repress the oncogenic miR-375 targets JAK2, YAP, PDK1 and RASD1 [46], [47], [48], [49]. Epigenetics have been implied in miR-375 regulation in breast cancer cells, where local depletion of DNA methylation and H3K9me2 and dissociation of the transcriptional repressor CTCF leads to miR-375 upregulation and subsequent activation of estrogen receptor α [47]. However, miR-375 silencing does not appear to be associated with DNA hypermethylation in OSCC (Fig. 2g).

As miR-375 silencing has been linked to a number of cancer types and target oncogenes, it probably acts as a general tumor suppressor miRNA. Although based on a small sample panel, this is, to our knowledge, the first report of altered miR-375 levels in body fluids of cancer patients. Clinical detection of repressed miR-375 levels may therefore not be limited to oral fluids and OSCC diagnosis. It would be interesting to investigate miR-375 presence in other bodily fluids, such as plasma or urine, for various cancer forms in order to establish a possible general diagnostic significance.

MiR-200a was also present at lower levels in oral rinse from cancer patients compared to healthy controls, but remained unchanged in saliva (Fig. 5b). Thus, oral rinse and saliva may have different value in clinical detection, and both types of oral fluid should probably be investigated independently when assessing diagnostic potential. It is possible that the greater exposure of the oral surface exfoliative buccal epithelia to environmental factors, such as for instance tobacco, makes these cells more prone to genetic and epigenetic aberrations. This suggests that oral rinse is a more sensitive source for clinical detection of OSCC specific miRNA and DNA methylation patterns than saliva, but this notion must be investigated in further detail.

Moreover, we confirm the previous reports of *miR-137* CpG hypermethylation in OSCC [7], [20]. Although statistically significant, this increase was small in the primary samples (∼15%, Fig. 2f), but in the CD44 sorted pure cell populations *miR-137* went from nearly fully methylated in cancer to unmethylated in normal, with corresponding expression patterns of the mature miRNA (Fig. 4c–d). However, contrary to Langevin *et al.* [21], we did not detect increased *miR-137* methylation in OSCC patient saliva or oral rinse (data not shown). An epigenetically independent increase in miR-137 was observed in CD44^high^ CSCs compared CD44^low^ cells, consistent with a role in differentiation [41], [42]. This demonstrates that miR-137 must be regulated by other mechanisms as well, which could explain why miR-137 remains undetected throughout the primary sample panel regardless of low DNA methylation in normal tissues (Fig. 2f).

MiR-127 was one of the first reported epigenetically regulated miRNAs [50], but this does not appear to relate to miR-127 changes in OSCC. Rather, we observed highly variable expression of this miRNA, associated with unchanged (adjacent normal vs. healthy epithelium) or anti-correlated (tumor vs. adjacent) loss of DNA methylation (Figs. 2b & 2h). Overall, miR-127 expression was elevated in patient tissues compared to healthy epithelium, suggesting a putative oncogenic role in OSCC. Moreover, miR-127 expression, but not methylation, was much higher in healthy stroma than epithelium, supporting that this miRNA normally functions in oral connective tissues and is aberrantly activated in squamous cell tumors (Figs. 2b & 2h).

In line with previous work by us and others in bladder, prostate and breast cancer [34], [35], [36], the miR-200 family and miR-205 are coordinately regulated in both transformed and healthy oral tissues. The miR-200s and miR-205 were also clearly epithelium specific, and epigenetic silencing appears to be a normal event preventing their expression in healthy stroma (Figs. 2d–f & 3c–d) [30], [31]. Consistent with a previous report of miRNA expression patterns in squamous head and neck cancer cell lines [51], we also observe that the miR-200 family and miR-205 are highly expressed in four oral squamous cell lines (SCC9, SCC25, SCC71 and CAL27; data not shown). However, there are indications that these miRNAs are comparatively repressed in poor prognosis disease [52]. Here, we observe miR-200/miR-205 activation in association with DNA hypomethylation in OSCC tumors compared to matched adjacent epithelium, but no obvious change between metastatic and non-metastatic tumors. Previous reports have shown that E-cadherin is expressed in OSCC, but fails to localize to the plasma membrane [53]. Hence, as opposed to other cancer forms, miR-200 activation of E-cadherin expression may not direct correct E-cadherin function in OSCC. A recent study showed that the miR-200s can also promote metastatic colonization through an E-cadherin independent pathway via direct targeting of Sec23a [54]. As such, these tumors may be highly aggressive and invasive despite elevated miR-200 expression in the tumor mass.

The specific silencing of the miR-200 family and miR-205 in CD44^high^ oral CSCs was not associated with increased DNA methylation. Thus, other mechanisms are probably initiating the primary repression of these miRNAs, with epigenetic silencing being a secondary and more definitive event in the tumor mass (Fig. 5a–b). The primary event could potentially be linked to EMT specific transcription factors such as ZEB, Twist, Snail or Slug, which have previously been associated with miR-200 regulation [55]. Given their role in maintaining E-cadherin expression and repressing Notch signaling, this primary silencing of miR-200/miR-205 is probably important for acquiring the dedifferentiated and migratory properties of tumor driving CSCs [35], [38]. Indeed, the miR-200 family and miR-205 are known to play complicated roles in malignant transformation. They appear to be activated during the transformation from normal tissue - to premalignant lesion - to carcinoma *in situ* in order to maintain epithelial properties, but then becomes repressed during mesenchymal transition, which drives invasion and metastasis [32]. Specific miR-200/miR-205 silencing in mesencymal-like oral CSCs, may thus be an important driver of tumor invasion and recurrence. The underlying mechanism of miR-200 and miR-205 deregulation may therefore provide powerful complementary therapeutic cancer targets.

### Conclusions

In summary, we report novel candidate OSCC deregulated miRNAs with associated DNA methylation patterns, and normal miRNA expression in healthy oral epithelium and stroma. MiR-375 is repressed and miR-127 activated in OSCC tumors, and, consistent with previous studies, we found increased DNA methylation of the *miR-137* locus in OSCC patients. However, miR-137 was below the detection limit in our assays, and FACS sorting of pure cell populations from patients and normal controls was required to observe distinct expression and methylation changes. The miR-200 family and miR-205 are overall epigenetically activated in OSCC tumors, but this may only reflect the tumor subtype. Rather, we speculate that epigenetically independent miR-200/miR-205 repression in oral CD44^high^ cells is an important event required for oral CSC formation and tumor progression. Several lines of evidence points to this scenario, but further work will be required to elucidate the complete regulatory mechanisms controlling regulation and function of this important miRNA family. Finally, we show that OSCC specific miRNA and DNA methylation patterns can be detected and distinguish between patient oral rinse and saliva. These findings provide a proof-of-concept, but larger randomized screens are required in the future to determine how applicable miRNA profiles and DNA methylation patterns in oral fluids will be as diagnostic predictors in clinical settings.

## Supporting Information

Figure S1 **Consistent expression of RNU48 and RNU44 among samples from both patients with oral carcinoma and healthy volunteers.** MiRNA expression profiling was performed using TaqMan® TLDA and included ncRNA reference genes RNU48 and RNU44. The CT value was presented for the amplification of RNU48 (Red) and RNU44 (Blue) among samples from different groups, including 2 non-metastatic tumors (18110T & 11063T), 2 metastatic tumors (17093T & 28169T), 4 matched adjacent normal tissues (18110N, 11063N, 17093N & 28169N), 2 healthy connective tissue (stroma) (H14C & H5C) and 2 healthy epithelium (H14E & H5E), respectively.
(TIFF)
