# Supplementary figures and images for: MicroRNA Alterations and Associated Aberrant DNA Methylation Patterns across Multiple Sample Types in Oral Squamous Cell Carcinoma
Source: PLoS One. 2011 Nov 22;6(11):e27840. doi: 10.1371/journal.pone.0027840 (PMC3222641; doi:10.1371/journal.pone.0027840)

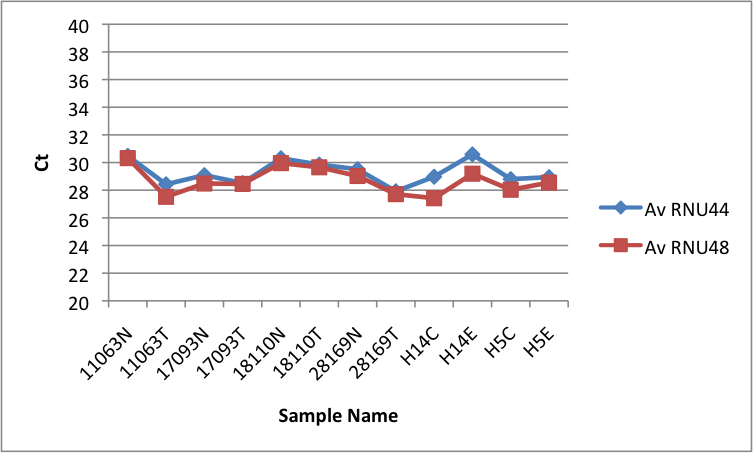

Supplement: Figure S1 — Consistent expression of RNU48 and RNU44 among samples from both patients with oral carcinoma and healthy volunteers. MiRNA expression profiling was performed using TaqMan® TLDA and included ncRNA reference genes RNU48 and RNU44. The CT value was presented for the amplification of RNU48 (Red) and RNU44 (Blue) among samples from different groups, including 2 non-metastatic tumors (18110T & 11063T), 2 metastatic tumors (17093T & 28169T), 4 matched adjacent normal tissues (18110N, 11063N, 17093N & 28169N), 2 healthy connective tissue (stroma) (H14C & H5C) and 2 healthy epithelium (H14E & H5E), respectively. (TIFF) [file pone.0027840.s001.tiff]
